# Selection of high temperature and salinity tolerant *Trichoderma* isolates with antagonistic activity against *Sclerotium rolfsii*

**DOI:** 10.1186/2193-1801-3-641

**Published:** 2014-10-29

**Authors:** Sowmya Poosapati, Prasad Durga Ravulapalli, Navaneetha Tippirishetty, Dinesh Kumar Vishwanathaswamy, Sarada Chunduri

**Affiliations:** Department of Plant Pathology, Directorate of Oilseeds Research, Rajendranagar, Hyderabad, 500030 India; Department of Biotechnology, Directorate of Oilseeds Research, Rajendranagar, Hyderabad, 500030 India; Department of Agricultural Statistics, Directorate of Oilseeds Research, Rajendranagar, Hyderabad, 500030 India

**Keywords:** *Trichoderma*, Thermotolerance, Saline tolerance, Trehalose, Mannose, Biocontrol, *Sclerotium rolfsii*

## Abstract

*Trichoderma* isolates were collected from varied agro-climatic zones of India and screened for high temperature and salinity tolerance. Among all the isolates tested, *T. asperellum,* TaDOR673 was highly tolerant to heat shock of 52°C with a mean spore count (log c.f.u/ml) of 4.33. The isolate after recovery from heat shock possessed higher germination rate and biomass production compared to its wild counterpart, upon prolonged exposure to 37°C. Under stress, TaDOR673 accumulated >15% of trehalose and >5% of mannose and raffinose compared to the wild type strain signifying their role in stress tolerance. *T. asperellum*, TaDOR693 and *T. asperellum*, TaDORS3 were identified as superior salt-tolerant isolates. Interestingly, TaDOR673 also possessed similar tolerance levels to increasing saline concentrations as indicated by its improved colony growth under stress conditions. *T. asperellum,* TaDOR673 and *T. asperellum,* TaDOR7316 effectively controlled the collar rot disease in groundnut by 79.7% when screened *in vitro* and *in vivo*. Thus, the study identified a potential thermotolerant and saline tolerant strain of *Trichoderma*, TaDOR673 that could be used as potential bioagent in stressed soils.

## Background

The fungi belonging to the genus- *Trichoderma* are in current use as biocontrol agents owing to their ability to antagonize other fungi (Howell 2003). The antifungal properties of *Trichoderma* are attributed to their ability to produce antibiotics (Vinale et al. 2008) or/and hydrolytic enzymes (Benitez et al. 2004) and competition for nutrients (Elad 2000). *Trichoderma* species are known to promote plant growth and induce biotic and abiotic stress resistance in plants (Djonovic et al. 2007; Harman et al. 2004). However the ability of these fungi to sense, invade and destroy other fungi has been the major driving force behind their commercial success as biopesticides and more than 60% of all registered biopesticides are *Trichoderma*-based (Verma et al. 2007).

Most of the *Trichoderma* strains are mesophilic and the competitive colonization of strains *viz*., *T. harzianum* was greatest at degrees lower than the temperature optimum for growth *in vitro* and its saprophytic activity was greatest from 15°C to 21°C (Eastburn and Butler 1991). However, adapted to virtually every ecosystem, these fungi live in marine and terrestrial sites (Johnson et al. 1987) and play an important role in ecosystem health (Klein and Eveleigh 1998). Gal-Hemed et al. 2011 isolated two potential halotolerant *Trichoderma* strains from Mediterranean sponges that reduced the damping off disease on beans caused by *Rhizoctonia solani* and also induced defense responses in cucumber seedlings against *Pseudomonas syringae* pv. *lachrimans*. The genus *Trichoderma*, is thus a rich source, to explore potential bioagents for application across adverse climatic zones.

In certain conditions, the performance of the *Trichoderma* strains as biocontrol agents is limited due to their low osmotolerance levels (Magan 1988) and such strains fail to protect the germinating seeds from soil-borne diseases caused by pathogens that tolerate high salt concentrations (El-Abyad et al. 1988; Ragazzi et al. 1994). The choice of bioagents is thus highly influenced by the soil hydrological parameters since they play a crucial role in monitoring *Trichoderma* activities especially, spore germination, germ tube growth (Magan 1988), mycelial growth (Luard and Griffin 1981) and antagonistic properties (Tronsmo and Dennis 1978). The effect of soil temperature on the radial extension of *Trichoderma* was also well evaluated (Knudsen and Bin 1990).

To date, research has mostly focused on identification of psychrophilic and salinity tolerant strains of *Trichoderma* and very few reports are available on high temperature tolerant *Trichoderma* that are effective at temperature above 35°C (Harman et al. 1981). Although soils in temperate regions during seeding stage are not often at this temperature, there is a need to identify potential high temperature tolerant *Trichoderma* strains. Such strains could sustain and survive the fluctuating temperatures rising due to global warming and naturally antagonize the sclerotia forming post-harvest pathogens *viz*., *Sclerotium rolfsii* that survive such adverse conditions in soils (Mukherjee and Raghu 1997). Better understanding of the genomics of such strains will improve and expand their applications.

Research groups have used different strategies to improve the *Trichoderma* strains, either by isolating the mutant strains of *Trichoderma* that possess better growth and bioefficacy in comparison to wild type strains (Mohamed and Haggag 2006) or by isolating naturally occurring potential antagonistic isolates (Nikolajeva et al. 2012). In our study, natural selection strategy was adopted in view that the isolates identified by this strategy would be biologically more stable and can easily be commercialized for field use.

In the present investigation, high temperature and salinity tolerant *Trichoderma* strains were isolated from natural soils and their biocontrol efficacy was evaluated. The study also focused on the biochemical estimation of stress protectants and polyols that are known to confer stress tolerance in fungi and plants (Pennycooke et al. 2004; Ruijter et al. 2003; Fillinger et al. 2001; Pedreschi et al. 1997). As studies have reported the effect of culture age on high temperature tolerance of fungi (Arif Mahmud and Ohmasa 2008), measures were taken to identify the optimum culture age of the stress-tolerant isolates identified in the study. The data thus obtained will help in the successful formulation and application of these bioagents in varied climatic zones and also will aid as a source in revealing the secret pathways conferring stress tolerance in *Trichoderma* species.

## Results

### Growth in relation to temperature stress

640 soil samples were collected from various agroclimatic zones of India and 250 isolates of *Trichoderma* isolated from them were morphologically characterized. The thermotolerant isolates identified from the stress tolerance studies were characterized by sequencing a section of elongation factor 1 alpha gene. According to NCBI BLAST search against the GenBank sequence database and comparison using TrichoBLAST and TrichoKey tools (Druzhinina et al. 2005), most of the isolates were identified as *T. asperellum* and their corresponding accession numbers were obtained from the NCBI. The geographical location of these isolates was also listed for reference (Table 1).

**Table 1:** **GenBank accessions of thermotolerant isolates of** ***Trichoderma*** **with their respective geographical location**

Isolate	Organism	Gene bank accession	Location	Crop under cultivation at the time of sample collection
TaDOR7316	*Trichoderma asperellum*	KM190858	Rajasthan	Soyabean
TaDOR224	*Trichoderma asperellum*	KM190853	Belgaum	Jowar
TaDOR33	*Trichoderma asperellum*	KM190854	Dharwad	Safflower
TaDOR293	*Trichoderma asperellum*	KM190857	Belgaum	Jowar
TaDOR294	*Trichoderma asperellum*	KM190852	Belgaum	Jowar
TaDOR671	*Trichoderma harzianum*	KM190861	Guntur	Cotton
TaDOR79	*Trichoderma asperellum*	KM190856	Rajasthan	Groundnut
TaDOR222	*Trichoderma asperellum*	KM190855	Rajasthan	Jowar
TaDORS3	*Trichoderma asperellum*	KM435275	Andhra Pradesh	Jowar
TaDOR564	*Trichoderma asperellum*	KM435274	Rajasthan	Cotton
TaDOR693	*Trichoderma harzianum*	KM190862	Rajasthan	Sesame
TaDOR712	*Trichoderma harzianum*	KM190860	Rajasthan	Pigeonpea
TaDOR673	*Trichoderma longibrachiatum*	KM190859	Rajasthan	Sorghum

Under the conditions of the conidial thermotolerance study, a comparison of the survival rates of the *Trichoderma* strains under heat stress was carried out. Lethal temperature 50 (LT_50_) was determined by screening the isolates at 48°C, 50°C and 52°C for 1 h, 2 h and 4 h, respectively, using probit analysis. Thirteen isolates were able to germinate significantly at higher temperatures and the c.f.u data were subjected to ANOVA analysis (Table 2). Repeated measures ANOVA showed that isolates (F_12, 117_ = 289.36, P <0.0001), temperature regimes (F_2, 117_ = 7603.6, P <0.0001) and time period (F_2, 117_ = 4763.23, P <0.0001) were significantly different from each other. The two interactions i.e., isolate by temperature regime (F_24, 117_ = 30.59, P <0.0001) and isolate by time (F_24, 117_ = 23.51, P <0.0001) and the three way interactions *viz*., isolate by temperature regime by time has shown significant difference (F5_2, 117_ = 39.77, P <0.0001). Mean spore count (c.f.u) was higher at 48°C (4.94) and gradually decreased with increase in temperature to a mean spore count of 3.38 at 50°C and 1.55 at 52°C, respectively.

**Table 2:** **Effect of temperature on the conidial germination determined by colony forming units (c.f.u)**

Isolate	Log cfu/ml at different temperatures and incubation periods												Grand mean
	48°C				50°C				52°C				
	1 h	2 h	4 h	Mean	1 h	2 h	4 h	Mean	1 h	2 h	4 h	Mean	
*T. asperellum*, TaDOR79	6.32^a^	5.70^b^	3.83^cd^	5.28^bc^	5.30^bc^	3.00^e^	1.50^d^	3.27^c^	2.84^c^	1.39^cd^	1.73^b^	1.99^b^	3.51^b^
*T. asperellum*, TaDOR33	6.45^a^	5.64^b^	3.15^fg^	5.08^de^	5.30^bc^	3.15^de^	1.28^de^	3.24^c^	2.39^de^	1.69^bc^	0.15^d^	1.41^ef^	3.24^cd^
*T. asperellum*, TaDOR7316	5.88^b^	4.90^df^	2.00^h^	4.26^h^	4.76^de^	3.15^de^	2.66^b^	3.52^b^	2.28^df^	0.85^e^	0.35^d^	1.16^gh^	2.98^e^
*T. harzianum*, TaDOR693	6.46^a^	5.17^cd^	3.15^fg^	4.93^ef^	5.06^cd^	4.45^b^	1.28^de^	3.60^b^	2.53^d^	1.94^b^	0.00^d^	1.49^ef^	3.34^c^
*T. harzianum,* TaDOR671	6.58^a^	5.70^b^	4.15^bc^	5.47^b^	4.45^f^	4.39^b^	0.00^g^	2.95^d^	2.23^efg^	1.24^d^	1.13^c^	1.53^de^	3.32^cd^
*T. asperellum,* TaDOR564	5.92^b^	5.47^bc^	4.32^b^	5.23^cd^	5.98^a^	4.24^bc^	0.50^f^	3.57^b^	2.27^df^	1.96^b^	0.89^c^	1.71^cd^	3.50^b^
*T. asperellum*, TaDOR294	5.84^b^	5.70^b^	3.80^cd^	5.11^ce^	5.38^b^	4.06^c^	0.00^g^	3.15^c^	3.43^b^	1.15^de^	0.00^d^	1.53^de^	3.26^cd^
*T. asperellum*, TaDOR224	6.41^a^	5.09^de^	2.82^g^	4.77^fg^	5.37^b^	3.24^de^	1.95^c^	3.52^b^	1.95^g^	1.24^d^	0.78^c^	1.32^fg^	3.20^d^
*T. asperellum,* TaDOR222	5.15^c^	4.84^df^	1.95^h^	3.98^i^	5.38^b^	3.15^de^	0.00^g^	2.84^d^	1.08^h^	0.00^f^	0.00^d^	0.36^i^	2.39^f^
*T. longibrachiatum*, TaDOR673	6.55^a^	6.36^a^	5.28^a^	6.06^a^	6.03^a^	5.41^a^	4.57^a^	5.34^a^	5.57^a^	4.04^a^	3.39^a^	4.33^a^	5.24^a^
*T. asperellum,* TaDOR293	6.30^a^	4.59^f^	3.01f^g^	4.63^g^	5.41^b^	3.45^d^	1.95^c^	3.60^b^	2.90^c^	1.45^cd^	0.93^c^	1.76^c^	3.33^c^
*T. harzianum,* TaDOR712	5.88^b^	4.78^ef^	3.28^ef^	4.64^g^	3.00^g^	2.00^f^	1.45^d^	2.15^e^	1.02^h^	0.15^f^	0.00^d^	0.39^i^	2.39^f^
*T. asperellum,* TaDORS3	5.78^b^	4.89^df^	3.54^de^	4.74^fg^	4.59^ef^	3.99^c^	0.95^e^	3.18^c^	2.08^fg^	1.15^de^	0.15^d^	1.13^h^	3.01^e^
LSD at α = 0.05	0.301	0.330	0.383	0.197	0.301	0.330	0.383	0.197	0.301	0.330	0.383	0.197	0.113
Mean	6.11	5.29	3.40	4.94	5.08	3.67	1.39	3.38	2.50	1.40	0.73	1.55	3.29

The values in the table represent the log10 transformation of spore concentration determined by c.f.u. The similar alphabets depicted in superscript in the columns indicate no significance difference in mean spore count (c.f.u).

**Figure 1:**
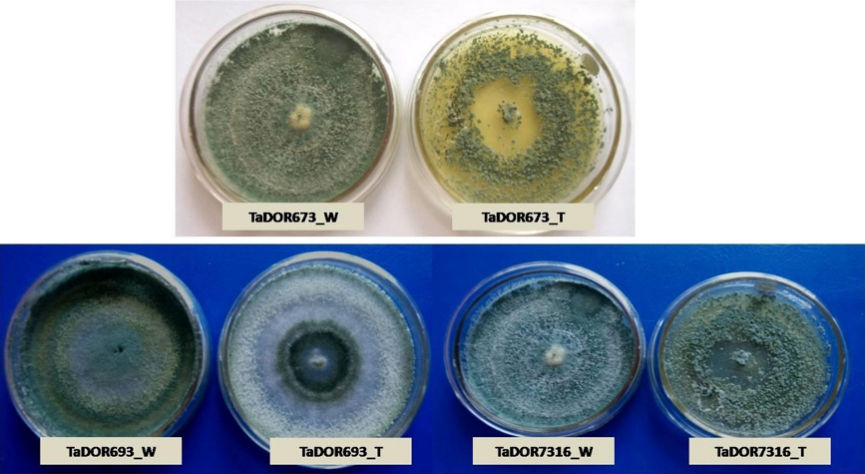
**Heat induced morphological changes in thermotolerant isolates of** ***Trichoderma*** **.**

From the analysis it was observed that *T. asperellum,* TaDOR673 was significantly different from all other isolates under all the tested conditions. The isolate was able to tolerate the highest temperature for higher durations. Although at higher temperatures there is reduction in mean spore count as time progresses, isolates *viz*., *T. asperellum,* TaDOR79, *T. harzianum,* TaDOR671, *T. asperellum*, TaDOR293 and *T. asperellum*, TaDOR564 were also able to survive the adverse conditions by producing considerable number of c.f.u (Table 2). After the heat shock the fungal colonies from the selected isolates were revived on PDA. The heat induced morphological characteristics were observed in the revived isolates. Most of the isolates had reduced sporulation, yellow pigmentation and increased hyphal growth. In order to select superior stress tolerant strains, the revived isolates were used for further thermotolerance screening against their wild type counterparts (Figure 1).

### Hyphal thermotolerance test

**Figure 2:**
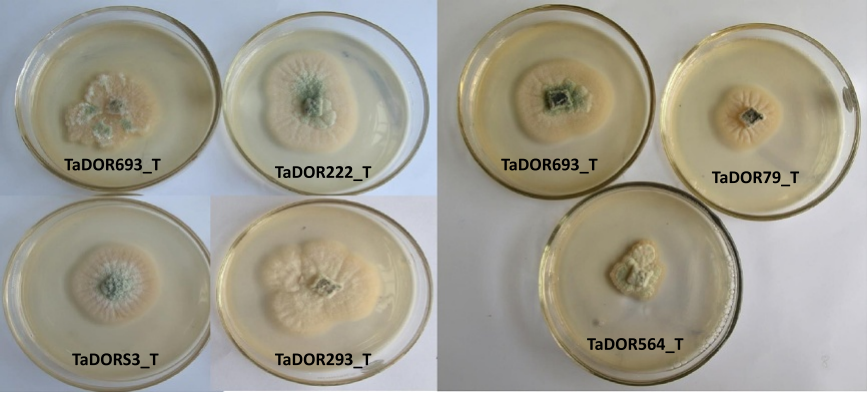
**Colony growth of thermotolerant isolates of** ***Trichoderma*** **at 35°C on potato dextrose agar media.**

**Figure 3:**
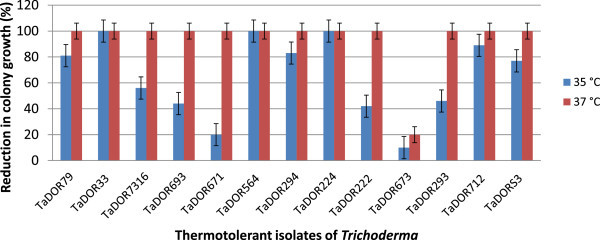
**Reduction in hyphal growth of thermotolerant isolates of** ***Trichoderma*** **in solid media.**

**Figure 4:**
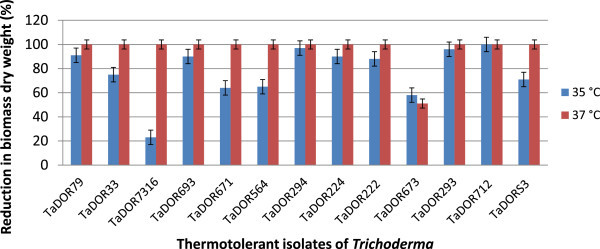
**Reduction in hyphal growth of thermotolerant isolates of** ***Trichoderma*** **in liquid media.**

**Figure 5:**
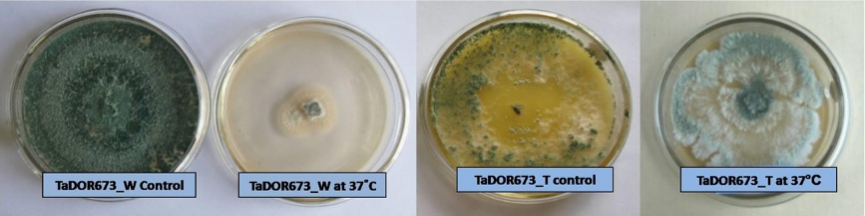
**Comparison of colony growth of wild type and thermotolerant isolate** ***T. asperellum*** **, TaDOR673.**

The growth rates of the thermotolerant isolates varied among each other and also with the media (solid/liquid) used in the study. At 35°C most of isolates could not germinate (Figure 2); however, the isolates *T. asperellum,* TaDOR673, *T. asperellum,* TaDOR7316 and *T. harzianum*, TaDOR671 could better survive the stress in comparison to their wild counterparts (Figures 3 and 4). With increase of temperature to 37°C the germination rates of all the isolates except for the *T. asperellum*, TaDOR673 was drastically reduced irrespective of the growth media (Figure 5). Furthermore when the hyphal cultures devoid of any spores was exposed to heat stress at 52°C for 4 h only *T. asperellum*, TaDOR673 was able to survive and germinate normally at 28°C indicating the ability of the isolate to survive adverse stress conditions.

### Salt stress of thermotolerant *Trichoderma*

Increasing saline concentrations of agricultural soils is an emerging concern that highly impacts the microbial ecosystem of the soil and its symbiotic interaction with the crop under cultivation. The thermotolerant isolates were thus further screened for their osmotolerance by exposing them to increasing concentrations of NaCl. All the isolates could survive better under saline stress in comparison to the heat stress. At 1 M NaCl concentration the germination rates of most of the isolates was reduced to 60-70%.

Repeated measure ANOVA revealed that colony growth (radii in cm) is highly dependent on nature of the isolates (F _12, 104_ = 327.38; P <0.001), salt concentration (F_3, 104_ = 887.38; P <0.001), and period/ time of culture (F _1, 104_ = 217.56; P <0.001). The mean colony growth (radii) was higher for *T. asperellum*, TaDOR693 (3.75 cm). Pair wise mean comparison with LSD at 5 per cent level of significance (0.194) illustrated that *T. asperellum*, TaDOR693; *T. asperellum*, TaDOR673; *T. asperellum*, TaDOR33 and *T. asperellum*, TaDORS3 were similar in growth compared to other isolates. Pair wise mean comparison (LSD = 0.108) also showed that effect of different salt concentrations on growth of isolates was significantly different from each other (LSD = 0.076).

**Figure 6:**
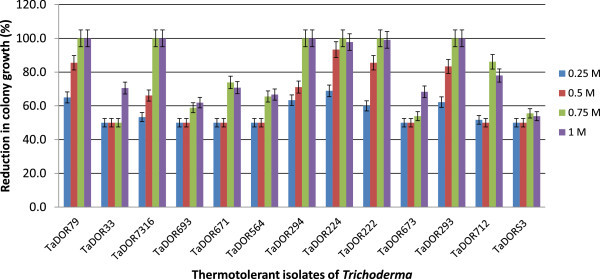
**Effect of high salt concentrations (NaCl) on colony growth of thermotolerant isolates of** ***Trichoderma*** **.**

At 0.5 M NaCl *T. asperellum*, TaDOR33 and *T. asperellum,* TaDOR224 were significantly tolerant with highest mean colony growth (radii = 4.48). At 0.75 M NaCl concentration 50% of the isolates lost their viability but the isolates, TaDORS3 (3.0 cm) and TaDOR693 (2.73 cm) had maximum colony growth at 1 M NaCl concentration indicating their high osmotolerance levels. Comparatively, the isolates *T. asperellum*, TaDOR673; *T. harzianum,* TaDOR671; *T. asperellum*, TaDOR564; *T. asperellum*, TaDOR693 and *T. asperellum*, TaDOR33 could sustain growth at higher saline concentrations and had 65-70% reduction in colony growth (Figure 6).

### Effect of culture age on thermotolerance

**Figure 7:**
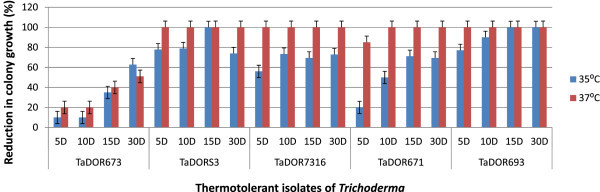
**Effect of culture age of thermotolerant isolates on colony growth at 35°C and 37°C.**

Factorial ANOVA has revealed the significant effects in the experiment *viz*., isolates (F _4, 39_ = 4099.58; P <0.001), age (F_3, 39_ = 39.39; P <0.001) and their interaction *viz*., age by isolate (F_12, 39_ = 39.39; P <0.001). Pair-wise comparison showed that mean colony growth of all the isolates was significantly different from each other. In all the tested isolates, the thermotolerance levels of the isolates were gradually reduced with increase in culture age of isolate. Moreover, the appropriate culture age for the thermotolerant isolates was different among each other i.e., for the *T. asperellum*, TaDOR673; *T. asperellum*, TaDOR7316, the appropriate culture age was between 5–10 days but for isolates *T. asperellum*, TaDORS3 and *T. asperellum*, TaDOR693 it was 15 days (Figure 7).

### Quantification of polyols under heat stress

**Figure 8:**
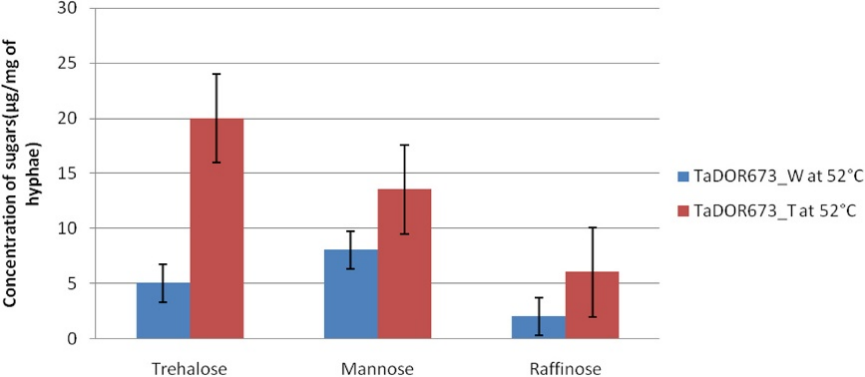
**Biochemical estimation of intracellular stress protectants in the thermotolerant isolate, TaDOR673.**

Thermotolerant isolates were exposed to 52°C for 1 h, 2 h and 4 h, respectively. The cell free extracts were subjected to HPLC for the estimation of sugars produced during the stress conditions. The metabolism of sugars *viz*., glucose, mannose, trehalose, melobiose tetrahydrate, raffinose and sucrose were higher in the stressed cultures. Upon exposure to heat stress there was increased production of known heat stress protectants like trehalose and mannose in thermotolerant isolates of *Trichoderma viz., T. asperellum*, TaDOR673 (Figure 8), *T. asperellum*, TaDOR79 and *T. asperellum,* TaDOR7316 as compared to their unstressed controls and wild type strains. There was a cyclic increase and decrease of trehalose and mannose concentration with increased exposure to heat stress. Moreover, increased accumulation of raffinose was also observed during the heat stress indicating its possible role in thermotolerance.

### Screening of selected superior stress tolerant isolates for biocontrol potential

**Figure 9:**
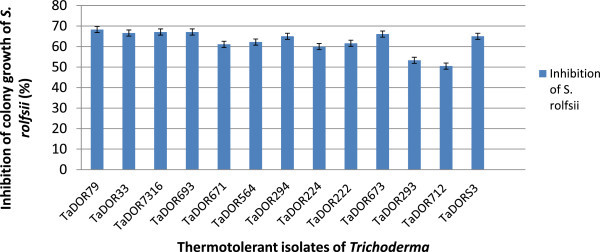
**Effect of thermotolerant isolates of** ***Trichoderma*** **on percent inhibition of radial growth of S. rolfsii in vitro.**

**Figure 10:**
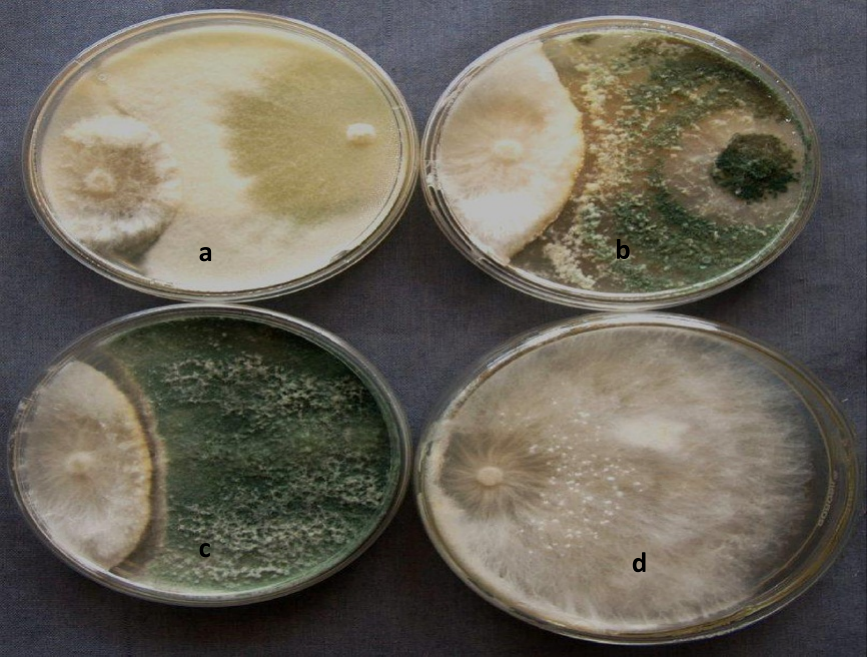
***In vitro*** **screening of thermotolerant isolates of** ***Trichoderma*** **against** ***S. Rolfsii*** **in dual culture plate method.** *a: TaDOR712; **b**: TaDOR673; **c**: TaDOR7316 against **d**: *S. Rolfsii*.

Most of the temperature tolerant isolates showed 50–68% reduction of hyphal growth of *S. rolfsii* when screened *in vitro* (Figures 9 and 10)*.* Of the 13 high temperature tolerant isolates that were screened *in vivo* against *S. rolfsii* in groundnut, 4 isolates *viz*., *T. asperellum*, TaDOR673; *T. asperellum*, TaDOR7316; *T. harzianum,* TaDOR671 and *T. asperellum*, TaDOR79 showed 70–80% reduction over controls (Table 3).

**Table 3:** ***In vivo*** **screening of thermotolerant isolates of** ***Trichoderma*** **against collar rot disease in groundnut**

Isolate	*Disease incidence (%)	Reduction over control (%)
*T. asperellum*, TaDOR7316	12.5 (15.3)	79.7
*T. asperellum,* TaDOR224	50.0 (45.0)	45.0
*T. harzianum,* TaDOR671	25.0 (22.8)	77.3
*T. asperellum*, TaDOR293	66.6 (54.7)	35.3
*T. asperellum*, TaDOR294	33.3 (35.2)	50.8
*T. asperellum*, TaDOR564	33.3 (35.2)	54.8
*T. asperellum*, TaDOR79	25.0 (22.8)	77.3
*T. asperellum*, TaDOR222	33.3 (35.2)	54.8
*T. asperellum*, TaDORS3	50.0 (45.0)	45.0
*T. asperellum*, TaDOR33	33.3 (35.2)	54.8
*T. harzianum*, TaDOR693	66.6 (54.7)	35.3
*T. longibrachiatum*, TaDOR673	12.5 (15.3)	79.7
*T. harzianum,* TaDOR712	66.6 (54.7)	35.3
SEM ±	3.1	
CD (P =0.05)	4.7	
CV (%)	10.2	

*Disease incidence recorded on 90th day after sowing; Values in parenthesis are arcsine transformation; Values are mean of 3 replications.

## Discussion

*Trichoderma* species have been widely used as potential biological control agents in commercial agriculture over the past 2–3 decades. Interestingly, the extracellular enzyme system of *Trichoderma* important for competition and mycoparasitism remains active even under environmental conditions unfavourable for mycelial growth and hence there is a possibility to improve the strains for better stress tolerance (Kredics et al. 2003).

Abiotic stresses are often interrelated and either individually or in combination, they cause morphological, physiological and molecular changes that adversely affect the growth of the organism. The efficient use of these bioagents across different agricultural soils and climatic conditions was highly limited by the soil hydrological factors (Magan 1988; Luard and Griffin 1981), with soil temperature being an important parameter affecting the radial growth and competitive colonization of the bioagents in the soil (Knudsen and Bin 1990). Several authors attempted to examine the influence of environmental parameters (water activity, temperature and pH) on the growth of *Trichoderma* and other fungal strains. Most naturally existing ascomycetes fungi have growth maximum at 30°C (Magan and Lacey 1984; Begoude et al. 2007) to 35°C (Moustafa and Abdel-Azeem 2008) but in contrast to those, the present investigation has identified some thermotolerant *Trichoderma* strains with highest growth rate at 37°C.

Under the conditions of conidial thermotolerant test, the isolate *T. asperellum*, TaDOR673 possessed the highest mean spore count of 3.39 (three way ANOVA analysis) followed by *T.* asperellum, TaDOR79 (1.73). *T. asperellum*, TaDOR673 was significantly different from other isolates in the study. There was gradual decrease of spore count (c.f.u) with increase in temperature and it dropped drastically at 52°C (1 h exposure). Different isolates had different germination rates at higher temperatures and after recovery from the heat stress most isolates had sparse sporulation, increased hyphae or yellow pigmentation. Among the thermotolerant isolates tested for improved thermotolerance, *T. asperellum*, TaDOR673 was identified as the superior stress tolerant isolate that survived prolonged exposures to heat stress at 37°C. This was clearly indicated by its increased germination rate (colony diameter) and biomass (dry weight). Moreover, when the hyphal cultures of thermotolerant isolates devoid of any spores were exposed to heat stress at 52°C for 4 h, only *T. asperellum*, TaDOR673 was able to survive and germinate normally at 28°C signifying its higher therrmotolerance with no significant influence on its growth parameters. Thus TaDOR673 is comparable and/or superior to other thermotolerant (Moretti Marcia et al. 2012) and mycoparasitic fungi (Yang et al. 2010) that are able to grow at 40-45°C.

Different *Trichoderma* isolates have different growth optima (Tronsmo and Dennis 1978) and identification of appropriate culture age aids in successful formulation of the bioagents. Conidial maturation stage, dependent on the media used for growth, is crucial for determining the thermotolerance levels of the isolates (Kim et al. 2010). The thermotolerant isolates identified in the study were highly active at a culture age of 5 to 10 days and after that there was decrease in the germination rate of the isolates under stress conditions.

At soil temperatures greater than 37°C the growth of fungi depends greatly on moisture levels and water activity acts as a crucial environmental factor that influences the radial growth of *Trichoderma* strains (Begoude et al. 2007). Moreover, low osmotolerance levels of *Trichoderma* isolates limits its boundaries for use as biofungicides (Kredics et al. 2003; Magan 1988; Luard and Griffin 1981). Thus, the present study also focused on the salinity tolerance of the selected thermotolerant isolates. All the isolates tested were comparatively more tolerant to saline conditions than higher temperatures. The statistical analysis revealed that *T. asperellum*, TaDOR693 and *T. asperellum*, TaDORS3 were highly tolerant to salt stress conditions. Moreover, thermotolerant isolate *T. asperellum*, TaDOR673 also showed significant tolerance to higher salt concentration (LSD = 0.521).

The higher thermotolerance of the isolate *T. asperellum*, TaDOR673 was attributed to its ability to accumulate stress protectants as evident from the HPLC study. Under stress conditions there was an increase in accumulation of trehalose, mannose and raffinose. These sugars were the most abundant polyols in the cells exposed to various stress conditions. Studies have shown that mannose constitutes 10–15% of dry weight of filamentous fungi and aids in abiotic stress tolerance (Ruijter et al. 2003). Our results were in accordance with the observation of Pedreschi et al. 1997, who observed the increased accumulation of trehalose in conidia of *Trichoderma harzianum* P1 on heat shock at 40°C for 90 min. The role of trehalose in fungal survival and as stabilizing agent of cell structures and cellular proteins under heat stress conditions was well investigated (Fillinger et al. 2001; Ruijter et al. 2003). Comparatively, *T. asperellum*, TaDOR673, *T. asperellum*, TaDOR79 and *T. asperellum,* TaDOR7316 were able to show greater metabolism of sugars like glucose, sucrose, raffinose and melobiose under control and stressed conditions, signifying their role as heat stress protectants. Dual culture and *in vivo* assay of the *Trichoderma* isolates against *S. rolfsii* were evaluated. *T. asperellum*, TaDOR673 and *T. asperellum*, TaDOR7316 were effective in controlling the plant pathogens with 79.7% reduction in disease incidence reported.

## Conclusion

From the study, two potential isolates of *Trichoderma viz*., *T. asperellum,* TaDOR673 and *T. asperellum,* TaDOR7316 were identified as tolerant to high salt and temperature conditions. The MTCC accession numbers for the isolates are MTCC 5622 & MTCC 5623, respectively. Provisional patent application was filed for patenting these stress tolerant *Trichoderma* isolates.

## Materials and methods

### Selective isolation of *Trichoderma*

Six hundred and forty soil samples from different regions of India were collected. The soil type was mainly sandy loam, clay, red and black soil. At the time of sample collection the crops under cultivation were groundnut, soybean, sorghum, bajra, cotton and sugarcane. Soil samples were serially diluted and plated on *Trichoderma* selective medium (TSM) (Elad et al. 1981; Askew and Laing 1993). Petri plates (90 mm diameter), were incubated at room temperature (26-30°C) for 7 days. *Trichoderma* isolates were selectively transferred onto potato dextrose agar (PDA) based on morphological characteristics (Rifai 1969; Watts et al. 1988). Stock cultures of the isolated *Trichoderma* strains were maintained at 4°C on potato dextrose agar (PDA) slants. To further characterize the thermotolerant isolates, a section of elongation factor 1 alpha gene was amplified using the primers EF1-728 F: 5′-CATCGAGAAGTTCGAGAAGG-3′ (Carbone and Kohn 1999) and TEF1R: 5′-GCCATCCTTGGGAGATACCAGC-3′ (Samuels et al. 2002). PCR reaction was performed with an initial 2 min at 94°C followed by 30 cycles of 30s at 94°C, 30s at 55°C and 1 min at 72°C, followed by a final 10 min at 72°C. Template DNA for sequencing was prepared directly from PCR products with the QIAquick PCR purification kit (Qiagen, Valencia, California). The sequences were subjected to BLAST in NCBI and also analysed using TrichoKey and TrichoBlast tools (Druzhinina et al. 2005) to obtain the identification of the isolates. After confirmation the sequences were submitted to NCBI GenBank.

### Growth and quantification of *Trichoderma*on media

Conidia of *Trichoderma* were grown on PDA at 28°C. Aerial conidia were harvested from 7–10 day old culture and the conidial suspension was filtered through three layers of cheesecloth. The filtrate was preserved at 4°C until use (Agosin et al. 1997). Spore concentration was measured as described by Norton and Harman, 1985 (Norton and Harman 1985) and a population density of 1 × 10^10^conidia/ml was used for all experiments.

### Effect of temperature on growth

The isolates of *Trichoderma* were subjected to different stress tolerance tests to identify superior stress tolerant *Trichoderma* strains.

### Conidial thermotolerance test

Initially to screen all the *Trichoderma* isolates collected, a conidial suspension of 1 × 10^10^ conidia/ ml was inoculated into vials containing 5 ml of potato dextrose broth (PDB). The isolates were exposed to heat shock at 48°C, 50°C and 52°C for 1 h, 2 h and 4 h durations (Plesofsky-Vig and Brambl 1985). Two replicates for each experimental condition were used and each experiment was repeated twice. After incubation, 1 ml of the culture from each vial was serially diluted and plated onto media selective for *Trichoderma*. The isolates incubated at ambient temperature (28°C) were treated as controls. The colony forming units (c.f.u) were measured for all the plates and the data was subjected to probit analysis for determination of lethal temperature 50 (LT_50_) (Throne et al. 1995). The revived colonies were transferred onto PDA plates and changes in morphological characteristics were recorded. These revived isolates hereafter mentioned as “thermotolerant isolates” were used to test their thermotolerance at higher temperatures for prolonged durations.

### Hyphal thermotolerance test

From the periphery of 7 day old cultures of thermotolerant isolates, mycelial discs were taken out by a cork borer (5 mm diameter) and placed at the center of the PDA plates in two replicates. The culture plates were incubated at 35°C and 37°C respectively for 7 days. Visualization of continuous hyphal growth from heat treated mycelial plugs were determined as viable or survival. The diameter of hyphal growth was measured and compared between the isolates. Alternatively the mycelial plugs were inoculated into 100 ml of potato dextrose broth (PDB) and incubated at 37°C at 200 rpm. After 7 days of incubation, the mycelial growth from each flask was filtered using cheesecloth and their respective dry weights were measured.

In another method, a mycelial disc of respective cultures was inoculated into 100 ml of potato dextrose broth in conical flasks (250 ml Erlenmeyer flask) and incubated overnight at 28°C and 200 rpm. The hyphal culture devoid of any spores (ensured by microscopic observation) was placed in a water bath at 52°C for 4 h. After incubation, culture suspension from respective cultures was plated onto TSM plates and incubated at 28°C. Appearance of hyphal growth was monitored and compared between thermotolerant isolates.

### Salt stress to thermotolerant *Trichoderma*strains

Mycelial discs of thermotolerant isolates of *Trichoderma* were placed onto PDA medium supplemented with 0.25 M, 0.5 M, 0.75 M and 1.0 M NaCl, respectively, and incubated at 28°C for 7 days. Degree of survival was determined by measuring the colony growth on the media. Two replicates for each concentration were used and the strains grown at optimum temperature were treated as control.

### Effect of culture age on thermotolerance

To determine the effect of culture age on thermotolerance, the thermotolerant isolates were cultured on PDA petri plates with four culture ages i.e., 5, 10, 15 and 30 days and incubated at 28°C. After incubation, mycelial discs from each culture plate were placed at the center of the PDA medium in petri dishes and incubated at 37°C for 7 days. Degree of heat stress tolerance was determined by measuring the colony growth.

### Quantification of polyols by HPLC

The intracellular polyols were estimated in thermotolerant isolates as described by Hallsworth and Magan, 1996 with little modifications. One week old cultures grown in 100 ml of potato dextrose broth were exposed to 52°C for 1 h, 2 h and 4 h in shaking water bath at 200 rpm. The wild type counterparts of thermotolerant isolates served as control. All the samples were harvested and frozen in liquid nitrogen. One milliliter of distilled water was added to 250 mg of ground mycelia powder and placed in boiling water bath for 5 min. The samples were centrifugation at 12,000 rpm for 15 min and supernatant was preserved at -20°C till use. Quantitative analysis was carried out using HPLC (Shimatzu Corp. Japan, SPD-10AVp) with a refractive index detector, an amino form column and acetonitrile: water (80:20) as a mobile phase. Twenty-μL of sample/standard were injected and chromatograhed at a constant flow rate of 1 ml/min (mobile phase). Standards of glucose, trehalose, fructose, sucrose, arabinose, xylose, mannose, rhamnose, raffinose, melobiose monohydrate and glycerol with concentrations ranging from 0.1 to 1% were used as standards and their respective retention times were identified. The concentrations of the sugars in treated samples were compared to their unstressed counterparts and were expressed as μg/mg of hyphae used for the analysis.

### *In vitro*and *in vivo*screening of thermotolerant isolates of *Trichoderma*

Selected thermotolerant isolates were screened *in vitro* against *Sclerotium rolfsii* isolates by dual-culture technique (Dennis and Wesbter 1971) on PDA plates. The percentage of inhibition of pathogen was calculated by measuring the radial growth of pathogen in both control and dual culture plates. For *in vivo* studies, *S. rolfsii* grown on sorghum grains and 30 g of this inoculum was used for infecting a kilogram of sterile soil in pots for 5 days. Surface disinfected seeds of groundnut were treated with conidial suspension of 7 day old thermotolerant isolates for 2 h and sown in pathogen sick soil pots. Seeds inoculated with pathogen alone served as pathogen check and five replicates for each treatment were used. An un-inoculated control was also maintained for comparison of plant growth promotion by the isolates. The studies were carried out under optimum greenhouse conditions with the unstressed isolates as controls. Percentage of disease incidence and growth parameters *viz*., shoot length, root length, vigor index and dry weight of biomass were recorded at 30, 45, 60 and 90 days after plant growth. The potential thermotolerant isolates identified, were deposited in Microbial Type Culture Collection & Gene Bank (MTCC), Chandigarh as per Budapest Treaty guidelines, for patenting the naturally modified stress tolerant isolates of *Trichoderma*.

### Statistical analysis

Repeated measures Analysis of Variance (ANOVA) were used to analyze spore count and percentage reduction of conidia over time. Pair-wise mean comparison was done based on Fisher’s Least Square Difference. All the analyses were done in SAS V9.2 using Proc Mixed, Proc GLM and means comparison was done with LSMEANS. A macro developed by Piepho 2012, was used to generate letter display for mean comparisons obtained from Proc Mixed output. For the biocontrol studies the statistical analysis of data recorded in pot culture experiment was subjected to ANOVA. The level of significance used was P = 0.05.
